# A benchmark dataset for ensemble framework by using nature inspired algorithms for the early-stage forest fire rescue

**DOI:** 10.1016/j.dib.2020.105686

**Published:** 2020-05-13

**Authors:** HongGuang Zhang, ZiHan Liang, HuaJian Liu, Rui Wang, YuanAn Liu

**Affiliations:** School of Electronic Engineering, Beijing Key Laboratory of Work Safety Intelligent Monitoring, Beijing University of Posts and Telecommunications, Beijing, China

**Keywords:** Forest fire suppression, Rescue ensemble, Forest fire rescue, Rescue simulator, Dynamic optimization problem, Unmanned platforms

## Abstract

This paper introduces a benchmark dataset to the research article entitled “Ensemble framework by using nature inspired algorithms for the early-stage forest fire rescue - a case study of dynamic optimization problems”, by Zhang et al. [7]. Rescue ensemble that consists of rescue simulator and rescue algorithm is characterized by supporting the dynamic simulation of forest fire rescue. The purpose of rescue algorithm is to minimize the longest flight time of aircraft group II and the newly-increased burnt forest cost in one period, simultaneously. The map information in our dataset is from Google map and relevant parameters are also from the actual situation data. The benchmark contains 10 different maps that researchers can use to evaluate their own algorithms and compare their performance with our algorithm.

Specifications Table

**Table:** 

Subject	Decision Sciences: Management Science and Operations Research
Specific subject area	Dynamic optimization research for forest fire rescue
Type of data	Table Graph
How data were acquired	From Google map and the actual situation data
Data format	Raw Analyzed
Parameters for data collection	It is necessary to collect data on the distribution of forests on different terrains. According to the distribution of vegetation, it is divided into four areas: no vegetation, sparse vegetation, normal vegetation, and dense vegetation.
Description of data collection	Firstly, 10 representative-terrain forests are identified. Secondly, these forests are found on Google map and cut into four areas according to the color depth: no vegetation, sparse vegetation, normal vegetation, and dense vegetation. Lastly, these terrain data is stored in 10 sheets. Other parameters are set manually according to the actual situation data.
Data source location	BaDaLing forest, White grass forest, MangShan forest, LongBeiShan forest, DuHe basin, XiJiang basin, HuaYu island, HuaShan mountain, and TaiShan mountain in China. Spetses island in Greece.
Data accessibility	With the article
Related research article	Zhang, H., Liang, Z., Liu, H., Wang, R., Liu, Y., 2020. Ensemble framework by using nature inspired algorithms for the early-stage forest fire rescue — A case study of dynamic optimization problems. Eng. Appl. Artif. Intell. 90, 103517. https://doi.org/10.1016/j.engappai.2020.103517.

## Value of the data

• The data provided in this paper contains 10 test problems from the real data of forest fire rescue, which can be used to simulate forest fire rescue and verify the effectiveness of the algorithms.
• The data presented in this paper can save time for researchers who need to use this data to simulate the performance of different types of optimization algorithms in forest-fire-rescue systems.
• In [7], rescue algorithms are to minimize the longest flight time of aircraft group II and the newly-increased burnt forest cost in one period, simultaneously. We give the performance comparisons of five algorithms based on the same dataset and the original data of this ensemble framework can be used to compare the relative performance of the optimization algorithms.
• The benchmarks can be used in different forest-fire-rescue systems and test the rescue performance of optimization algorithms.

## 1 Data Description

Our benchmarks contain 10 forest-fire-rescue problems, which could be downloaded from https://github.com/1654402787/BenchmarksRescueEnsemble. These problems are based on forest, basin, island, and mountain maps. Since the slope data (e.g. *E_1_* and *E_2_*) cannot be obtained, we assume that *E_1_* = *E_2_*. Thus, the fire propagation probability *p_s_* based on the slope is equal to 1. Our simulation is offline. Therefore, we assume that the time period of running rescue algorithm once *T_ra_* and the time period of running rescue simulator once *T_rs_* are fixed. Moreover, *T_ra_*=10s and *T_rs_*=20s. According to the actual helicopter data, the flight speed of each aircraft *V* = 50m/s and the maximum number of firefighting cells of each aircraft *FEC* = 8. The length and width of each cell *L* = 5m and cell's area = 25m^2^. The parameter setting and the vegetation density data of each benchmark are stored in the data files named Benchmarks*N*.mat and VegetationDensity_*N*.mat, where *N* denotes the index of benchmarks. The parameters in Benchmarks*N*.mat and VegetationDensity_*N*.mat are explained in Table 1.

**Table 1:** Parameters in Benchmarks*N*.mat and VegetationDensity_*N*.mat.

Parameters	Descriptions
*L*	The length and width of each cell (m).
*R*	The cell number of each row.
*C*	The cell number of each column.
*windDir*	The angle between north direction and wind direction (°).
*windSpeed*	The wind speed (m/s).
*initIgnitionPoint*	The position of initial ignition point.
*fireExtinguishBase*	The position of firefighting bases.
*noVegetation*	The mark of no vegetation cell in VegetationDensity_*N*.mat.
*sparsVegetation*	The mark of sparse vegetation density cell in VegetationDensity_*N*.mat.
*nomaVegetation*	The mark of normal vegetation density cell in VegetationDensity_*N*.mat.
*densVegetation*	The mark of dense vegetation density cell in VegetationDensity_*N*.mat.
*FireAngleVectorMap*	The fire propagation vector of each cell.
*WindAngleVectorMap*	The wind angle vector of each cell.

Five algorithms are adopted as rescue algorithms, which are CMA evolution strategy (CMA-ES) [2,5], spider monkey optimization (SMO) [1,4], league championship algorithm (LCA) [3], a self-adaptive artificial bee colony algorithm (SABC) [6], and the proposed firefighting particle swarm optimization (FPSO). Their parameters are given in [7] In particular, FPSO uses the common setting of *c_1_, c_2_*, and *w*. Besides, *V_lim_* is an important parameter to determine the performance of FPSO. Therefore, we provide the sensitivity analysis to explain the *V_lim_* setting in Table 2.

**Table 2:** Sensitivity analysis data to explain the *V_lim_* setting in FPSO by using the mean of 10-run *FT, RER*, and *BVR* data.

*V_lim_*	F1			F3			F5			F7			F9		
	*FT*(s)	*RER*(%)	*BVR*(%)	*FT*(s)	*RER*(%)	*BVR*(%)	*FT*(s)	*RER*(%)	*BVR*(%)	*FT*(s)	*RER*(%)	*BVR*(%)	*FT*(s)	*RER*(%)	*BVR*(%)
2	298	11.20	3.65	110	13.58	0.95	346	12.39	3.64	290	8.58	4.24	306	9.05	1.27
4	514	12.61	7.15	118	11.33	0.94	480	8.27	5.78	688	11.75	8.22	142	12.70	0.66
6	492	11.89	7.27	472	7.34	2.85	168	11.13	1.14	488	9.59	6.02	366	11.22	1.32
8	482	10.61	6.97	146	13.51	0.95	494	11.78	4.56	478	9.83	6.57	104	11.59	0.64
10	698	9.43	10.36	674	8.85	4.06	128	13.00	1.11	824	7.70	10.17	298	8.93	1.19
12	888	9.37	13.50	320	14.73	2.06	432	11.16	3.30	298	8.66	4.43	330	13.02	1.43
14	488	9.65	7.15	338	14.58	2.00	874	7.22	10.25	126	14.18	1.58	466	8.41	1.95
16	864	9.51	13.35	480	11.21	2.94	890	6.04	9.21	840	9.40	10.98	314	10.16	1.40

To compare with the key rescue effects, we provide four evaluation criterions, which are the offline error rate (µ) in Eq. (1), the forest-fire-rescue time (*FT*), the rescued edge ratio (*RER*) and the burned value ratio (*BVR*). Explanations are as follows. (i) In Eq. (1), *K* was the number of dynamic fitness landscape changes, *f_k_* was the optimal solution obtained by the algorithm from the *1*st fitness landscape to the *k*th fitness landscape, and *h_k_* was the optimal value of the algorithm in the *k*th fitness landscape. (ii) *FT* was equal to the number of performing rescue algorithm × *T_ra_*. (iii) *RER* was equal to the number of the total cells that have been put out in the edge after fire (*PutOutNumberInEdge*) / the number of the total burnt cells after fire (*BurntCellNumber*) in our map. (iv) *BVR* was equal to the cost of the total burned cells after fire / the total cost of cells in our map.

(1) $$\mu =\frac{1}{K}\sum_{k=1}^{K}\left|h_{k}-f_{k}\right|$$

Besides, Tables 3, 4, 5, and 6 provide the experiment data of the evaluation criterions.

**Table 3:** The mean value of the offline error rate µ for 30 runs to test the dynamic search performance of FPSO.

No.	CMA-ES	LCA	SMO	SABC	FPSO
F1	1.82E-02	1.42E-02	1.47E-02	1.46E-02	7.61E-03
F2	6.88E-03	5.95E-03	7.03E-03	5.42E-03	4.17E-03
F3	1.58E-02	1.76E-02	1.89E-02	1.76E-02	1.53E-02
F4	6.24E-03	3.95E-03	3.59E-03	5.05E-03	2.84E-03
F5	8.09E-03	1.09E-02	1.56E-02	8.70E-03	8.67E-03
F6	3.15E-02	3.37E-02	2.68E-02	3.25E-02	2.77E-02
F7	9.70E-03	1.04E-02	9.09E-03	9.99E-03	8.10E-03
F8	3.41E-03	3.29E-03	3.31E-03	3.46E-03	3.85E-03
F9	4.07E-02	4.94E-02	5.83E-02	4.42E-02	3.51E-02
F10	6.38E-03	4.46E-03	3.99E-03	5.60E-03	4.68E-03

**Table 4:** The mean value of *FTs* for 30 runs to illustrate the performance of FPSO in respect of putting out forest fire as soon as possible.

No.	CMA-ES	LCA	SMO	SABC	FPSO
F1	1812.67	1366.67	1497.33	1565.33	547.33
F2	488.67	466.00	722.00	348.00	94.67
F3	718.67	728.67	988.00	1320.67	196.00
F4	1382.00	796.00	622.00	1184.00	240.67
F5	341.33	601.33	1186.00	682.00	564.67
F6	397.33	424.67	352.67	802.00	88.00
F7	1150.00	783.33	764.67	1306.67	303.33
F8	577.33	360.00	140.67	716.67	64.67
F9	714.00	735.33	1622.00	1300.67	363.33
F10	936.67	667.33	461.33	1066.00	486.67

**Table 5:** *RER* data about the mean value and standard deviation for 30 runs to illustrate the performance of FPSO in respect of controlling the spread speed of forest fire edge as much as possible.

No.	CMA-ES		LCA		SMO		SABC		FPSO	
	Mean value	Standard deviation	Mean value	Standard deviation	Mean value	Standard deviation	Mean value	Standard deviation	Mean value	Standard deviation
F1	2.82	3.16	6.84	4.89	5.97	3.75	5.85	4.56	9.48	5.69
F2	9.03	5.93	9.60	5.53	6.66	4.59	8.81	4.88	10.60	4.14
F3	9.57	5.67	8.71	4.25	7.70	5.05	6.83	4.21	11.97	5.47
F4	5.54	5.82	7.81	5.83	9.36	5.86	5.58	5.48	11.31	5.59
F5	9.91	5.87	9.27	5.62	7.39	6.47	9.47	6.12	9.59	5.76
F6	7.41	3.20	9.01	4.41	9.21	5.29	8.05	5.35	7.78	3.80
F7	6.80	5.70	8.31	4.15	9.34	4.47	6.50	4.43	11.91	5.90
F8	6.41	3.73	8.12	3.90	7.64	3.32	5.69	3.26	8.96	3.32
F9	7.93	4.13	9.53	5.52	5.79	3.81	5.58	3.67	10.32	5.61
F10	7.56	7.24	6.81	5.37	10.66	6.38	5.90	5.63	8.57	6.80

**Table 6:** The mean value of *BVR* for 30 runs to illustrate the performance of FPSO in respect of controlling the cost of burned forest.

No.	CMA-ES	LCA	SMO	SABC	FPSO
F1	28.71	21.31	23.70	24.49	7.97
F2	4.51	4.35	7.03	3.21	0.61
F3	4.12	4.32	5.69	7.45	1.35
F4	16.82	8.98	6.76	13.37	2.27
F5	3.93	7.42	14.77	7.13	6.22
F6	3.02	3.44	2.42	6.28	0.78
F7	15.12	10.51	9.85	17.23	4.09
F8	7.18	4.96	2.93	8.64	2.27
F9	2.69	3.00	6.18	5.01	1.46
F10	11.37	7.83	4.33	12.97	4.47

## 2 Experimental Design, Materials, and Methods

To express the sensitivity analysis data concisely, we use F1, F3, F5, F7, and F9 as the representatives of our test problems. In Table 2, these data indicate that when *V_lim_* is 4, 6, and 8, FPSO is able to obtain better performance. Thus, *V_lim_* is set as 8 in [7].

Five rescue algorithms are used in dynamic forest fire rescue. The µ value is the offline error rate to demonstrate the dynamic search performance. The mean values of µ, *FT, RER*, and *BVR* are used to compare the various performances of FPSO in the dynamic task assignment.

Table 3 provides the mean value of µ for 30 runs. Except for F5, F6, F8, and F10, FPSO has the best performance. This indicates that FPSO is able to track the best solution, even in dynamic forest-fire-spread environments. Besides, to further demonstrate the dynamic search capability of FPSO, we use Wilcoxon test at 0.05 level of significance for the 30-run µ data sets of four algorithms, as shown in [7].

*FT* is one of the most important rescue indexes from a real rescue perspective. *FT* not only is directly proportional to the capability of controlling forest fire spread but also corresponds to reducing the risk that large-scale forest fires happen. Table 4 gives the mean value of *FTs* for 30 runs. Except for F5 and F10, FPSO has the best performance. This indicates that FPSO is more applicable to forest fire rescue system than other algorithms. Besides, to further demonstrate the FPSO performance in respect of putting out forest fire as soon as possible, we use Wilcoxon test at 0.05 level of significance and the data are given in [7].

Table 5 provides the mean value and standard deviation of *RERs* for 30 runs. Expect for F5, F6, and F10, *RER* data of FPSO are the best from a mean-value perspective. In F5 and F6, FPSO is the second. This shows that FPSO is more efficient than other algorithms in forest fire rescue.

Table 6 gives the mean value of *BVR* to illustrate the cost value of burned forest. We compute the mean value of *BVRs* for 30 runs. Except for F5 and F10, FPSO has the best performance. In F5 and F10, FPSO is the second. Besides, the *BVR* distributions of FPSO are closer to smaller values in most cases. This indicates that the FPSO's capability of controlling the burned-forest cost is satisfactory.

## References

[1] Bansal J.C., Sharma H., Jadon S.S., Clerc M. (2014). Spider monkey optimization algorithm for numerical optimization. Memet. Comput..

[2] Hansen N., Lozano J.A., Larrañaga P., Inza I., Bengoetxea E. (2006). The CMA evolution strategy: a comparing review. Towards a New Evolutionary Computation: Advances in the Estimation of Distribution Algorithms.

[3] Kashan A.H. (2014). League championship algorithm (LCA): an algorithm for global optimization inspired by sport championships. Appl. Soft Comput..

[4] Sharma A., Sharma H., Bhargava A., Sharma N., Bansal J.C. (2017). Optimal placement and sizing of capacitor using Limaçon inspired spider monkey optimization algorithm. Memet. Comput..

[5] Varelas K., Auger A., Brockhoff D., Hansen N., ElHara O.A., Semet Y., Kassab R., Barbaresco F., Auger A., Fonseca C., Lourenço N., Machado P., Paquete L., Whitley D. (2018). A comparative study of large-scale variants of CMA-ES.

[6] Xue Y., Jiang J., Zhao B., Ma T. (2018). A self-adaptive artificial bee colony algorithm based on global best for global optimization. Soft Comput..

[7] Zhang H., Liang Z., Liu H., Wang R., Liu Y. (2020). Ensemble framework by using nature inspired algorithms for the early-stage forest fire rescue — A case study of dynamic optimization problems. Eng. Appl. Artif. Intell..

